# Influence of different *Hypericum perforatum* L. preparations on pharmacokinetic and pharmacodynamic properties of pentobarbital, diazepam and paracetamol

**DOI:** 10.1186/2050-6511-13-S1-A11

**Published:** 2012-09-17

**Authors:** Aleksandar Rašković, Nebojša Stilinović, Momir Mikov

**Affiliations:** 1 Department of Pharmacology, Toxicology and Clinical Pharmacology, Faculty of Medicine, University of Novi Sad, 21000 Novi Sad, Serbia

## Background

Herb–drug interactions are an important safety concern and the study was conducted regarding the interaction between the natural top-selling antidepressant remedy *Hypericum perforatum* and conventional drugs.

## Methods

This study examined the influence of acute pretreatment with different *H. perforatum* extracts on pentobarbital-induced sleeping time impairment of motor coordination caused by diazepam and paracetamol pharmacokinetics in mice. The preparations profile of St. John’s wort was determined using RP-HPLC analysis. Ethanolic extract, aqueous extract, infusion, tablet and capsule of *H. perforatum* were used in the experiment.

## Results

By quantitative HPLC analysis of active principles, it has been proved that *H. perforatum* ethanolic extract has the largest content of naphtodianthrones: hypericin (57.8 µg/ml) and pseudohypericin (155.4 µg/ml). Pretreatment with ethanolic extract of *H. perforatum* potentiated the hypnotic effect of pentobarbital and the impairment of motor coordination caused by diazepam to the greatest extent and also increased the paracetamol plasma concentration in comparison to the control group. These results were in correlation to naphtodianthrones concentrations.

## Conclusions

The obtained results show a considerable influence of *H. perforatum* on pentobarbital and diazepam pharmacodynamics and paracetamol pharmacokinetics.

